# Preserving cortico-striatal function: deep brain stimulation in Huntington’s disease

**DOI:** 10.3389/fnsys.2015.00032

**Published:** 2015-03-11

**Authors:** Sean J. Nagel, Andre G. Machado, John T. Gale, Darlene A. Lobel, Mayur Pandya

**Affiliations:** ^1^Cleveland Clinic, Neurologic Institute, Center for Neurological RestorationCleveland, Ohio, USA; ^2^Department of Neurosurgery, Cleveland Clinic, Neurologic InstituteCleveland, Ohio, USA; ^3^Department of Neuroscience, Cleveland Clinic, Lerner Research InstituteCleveland, Ohio, USA; ^4^Department of Psychiatry, Cleveland Clinic, Neurologic InstituteCleveland, Ohio, USA

**Keywords:** deep brain stimulation, Huntington’s disease, globus pallidus, striatum, cognition, chorea

## Abstract

Huntington’s disease (HD) is an incurable neurodegenerative disease characterized by the triad of chorea, cognitive dysfunction and psychiatric disturbances. Since the discovery of the HD gene, the pathogenesis has been outlined, but to date a cure has not been found. Disease modifying therapies are needed desperately to improve function, alleviate suffering, and provide hope for symptomatic patients. Deep brain stimulation (DBS), a proven therapy for managing the symptoms of some neurodegenerative movement disorders, including Parkinson’s disease, has been reported as a palliative treatment in select cases of HD with debilitating chorea with variable success. New insights into the mechanism of action of DBS suggest it may have the potential to circumvent other manifestations of HD including cognitive deterioration. Furthermore, because DBS is already widely used, reversible, and has a risk profile that is relatively low, new studies can be initiated. In this article we contend that new clinical trials be considered to test the effects of DBS for HD.

## Preserving Cortico-Striatal Function: Deep Brain Stimulation in Huntington’s Disease

Few conditions in medicine present patients and their families the challenges of Huntington’s disease (HD). Because it is a heritable disease, the majority of individuals at risk are exposed to a turbulent environment with a parent diagnosed with HD and faced with the reality of suffering a similar fate. At-risk patients have the option to learn if they have the disease and then must confront the sad truth that the disease will end in neurologic disability and premature death. For those who elect not to be tested, the specter of developing symptomatic HD still looms large. This in part contributes to the increased risk of suicide during early stages and engenders fear and desperation that perpetuates itself with time as families search for answers (Meiser and Dunn, 2000). This psychological burden, coupled with the absence of any promising interventions, contributes to low predictive testing rates and the potential to adopt overly optimistic beliefs about one’s future, which at times may have undesirable consequences from a medical or economic standpoint (Oster et al., 2013).

It is estimated that 30,000 individuals in the United States alone have manifest symptoms of HD, with twice as many additional individuals yet to manifest symptoms. Extensive efforts on the part of researchers, emboldened by these unfortunate facts, identified the genetic basis of HD in the early 1990’s. Since then, the effort to find a cure has accelerated, although to date only one therapeutic agent has been approved for clinical use. Physicians are therefore left to consider off-label uses of existing therapies. Deep brain stimulation (DBS) has been used in HD as a treatment for disabling chorea but targeted stimulation may be a potential consideration to palliate symptoms of HD.

Several longitudinal studies have contributed to the current understanding of the natural history of HD (Paulsen et al., 2008; Ross and Tabrizi, 2011; Ross et al., 2014). The mean age of onset is 40 with an additional 20 years of life expected after the disease manifests (Tabrizi et al., 2011). Onset is defined with the emergence of motor symptoms. Chorea—abrupt, random, involuntary movements—is the hallmark of HD. These movements often intensify before being replaced with voluntary motor deficits. The cognitive features of HD consist of executive dysfunction, visuospatial dysfunction, cognitive slowing, and loss of mental flexibility. Evidence suggests that cognitive dysfunction may parallel (or precede) the emergence of motor deficits (Paulsen and Long, 2014). The psychiatric manifestations vary widely, ranging from mood disturbances to obsessional anxiety and, rarely, psychosis. Furthermore, limbic dysfunction, in the setting of cognitive inflexibility and poor insight, contributes to a ‘dysexecutive syndrome’, characterized by perseveration, apathy, impulsivity and aggression. Non-pharmacologic interventions may be helpful in early stages but tend to become less effective as the disease progresses. Tetrabenazine is the only FDA-approved agent for the treatment of the chorea. Psychiatric symptoms are managed similarly to the general population. Cognitive symptoms are typically resistant to intervention. Biomarkers that correlate to disease stages have not yet been defined, although promising considerations in premanifest individuals have been identified (Paulsen et al., 2014).

## Pathogenic Mechanisms of HD

HD exhibits autosomal dominant inheritance and is equally prevalent in men and woman. In those who inherit the mutation, a cytosine-adenosine-guanine (CAG) repeat expansion is added to the IT15 huntington (HTT) gene on the short arm of chromosome 4 (Raymond et al., 2011). Affected individuals with the mutated form have between 36 and 121 CAG repeats in the coding region that translate into a polyglutamine expansion. The age of onset of motor symptoms inversely correlates with the number of repeated sequences (Ross et al., 2014). In healthy persons, HTT encodes a protein (huntingtin) responsible for synaptic vesicular transit and other cellular functions that when absent leads to *in utero* death (Cepeda et al., 2007). For example, wild type huntingtin contributes to the cortical production of brain derived neurotrophic factor (BDNF) before the latter’s downstream support of striatal neurons (Zuccato et al., 2010). In those with the mutation, glutamine residues accumulate and the mutant HTT protein disrupts cellular signals and homeostasis decades before symptoms emerge. The mutation is detectable in nervous tissue diffusely; however, the disease is uniquely characterized by its expression in the medium spiny neurons (MSNs) found in the caudate, putamen and cortical pyramidal cells. The eventual death of these MSNs is the pathological hallmark of the structural damage demonstrated in HD (Georgiou-Karistianis et al., 2013). The precise cascade of events that leads to neurodegeneration is still largely unknown (Raymond et al., 2011).

MSNs account for 90% of all neurons in the striatum and are its only output source (Murer et al., 2002). There are two subtypes of MSNs that are differentiated by the peptides co-expressed; the receptor subtypes and the target to which they project. These GABAergic MSNs exert their effect via a direct or indirect pathway. MSNs in the indirect pathway (striaopallidal, D2 receptors) project to the external segment of the globus pallidus (GPe) and subthalamic nucleus (Cepeda et al., 2007). These indirect pathway neurons are the first cell population to succumb in HD and are implicated in the hyperkinesia that heralds the onset of the disease. MSNs in the direct pathway (striatonigral, D1 receptors) project to the internal segment of the globus pallidus (GPi) and the substantia nigra pars reticulatea (SNr; Cepeda et al., 2007). Degeneration of direct pathway MSNs, and the resulting loss of substance P, may contribute to late stage motor features characterized by impaired voluntary movement (Raymond et al., 2011). This transition from hyperkinesia to akinesia during the natural history of the disease may confound the results of interventions aimed at arresting or controlling chorea.

Impaired dopamine homeostasis is another consequence of the mutation that contributes to the impaired information processing from cortical inputs to the striatum (André et al., 2010). Dopaminergic neurons projecting from the substantia nigra pars compacta, and to a lesser degree the ventral tegmental area (VTA), to the dorsal striatum regulate glutamate sensitivity as well (dorsal circuit). Indirect pathway MSNs express D2 receptors whereas D1 receptors are more abundant in direct pathway MSNs. Degeneration of these nigrostriatal dopaminergic neurons are observed in HD and may contribute to late onset akinesia (André et al., 2010; Raymond et al., 2011). Conversely, presynaptic hyperactivation of the nigrostiatal pathway may elicit the characteristic chorea of early stage disease (André et al., 2010). Agents affecting dopamine (DA) transmission are used to modulate HD symptoms with some effect. DA may facilitate the “upstate” or depolarized state that enables encoding of specific motor tasks routed by the corticostriatal pathway (Murer et al., 2002).

The primary inputs to the MSNs are glutamatergic projections from the neocortex. Cortical neuron death is also observed in HD, especially in layers III, V and VI. The cell death is profound enough to be observed on gross pathological specimens and predates the onset of motor symptoms. One hypothesis is that excitotoxicity at the cortico-striatal synapses underpins HD (Cepeda et al., 2007). This is supported by evidence that increased release of glutamate from cortical projections, together with reduced uptake from the synaptic cleft by glial cells and enhanced striatal sensitivity to glutamate contributes to an amplified affect (Wójtowicz et al., 2013). In mouse models of HD, down-regulation of GLT1 has been demonstrated (Liévens et al., 2001). GLT1, a sodium-dependent glutamate transporter, ordinarily serves to remove extracellular glutamate and limit excitotoxicity. This down-regulation, together with NMDA-R hypersensitivity, increases intracellular calcium and induces an apoptotic cascade in HD.

Aspiny cholinergic, fast spiking, interneurons that co-express parvoalbumin are the main inhibitory neurons within the striatum (Russo et al., 2013). Theses interneurons are typically preserved until the late stages of the disease and are at least partly responsible for information processing and integration in the normal striatum (Raymond et al., 2011). Although, the death of the interneurons lags behind the MSNs, the dystonia that is especially pronounced in early onset and juvenile HD may be related to interneuron death (Reiner et al., 2013).

## Abnormal Electrophysiology in HD

Under normal conditions, the ventral, dorsolateral (motor) and dorsomedial (associative) striatum are both activated simultaneously during specific task learning (Williams and Eskandar, 2006; Gale et al., 2014; Thorn and Graybiel, 2014). As a behavior becomes habitual, the association between striatal components decouples and the ventral (Gale et al., 2014) and motor striatum emerges while the activity of the associative striatum, now redundant, recedes (Williams and Eskandar, 2006; Thorn and Graybiel, 2014). In the ventral striatum, the learning related facilitation of activity is thought to represent the increased expectation of reward for the execution of specific stimulus-motor behaviors (Gale et al., 2014). Thus, under normal conditions, it is thought that the ventral striatum provides the motivation to engage in reward obtaining behaviors. In HD, the depressive and limbic symptoms may be related to motivational changes brought on by dysfunction of striatal MSNs and/or dopamine de-innervation (via dopamine cell loss of the SNpc and VTA). In the dorsal striatum, once a task is learned, the specific motor sequences or chunks are organized within the basal ganglia as a concatenation of neuronal activity played out in the direct and indirect pathway. Co-activation of these pathways facilitates the desired movement by suppressing unwanted movements (indirect) and expressing the specific motor chunk (direct pathway) (Jin et al., 2014). Near limitless combinations of nested oscillating frequencies are able to associate with these specified action sequences. Normally, when an action is queued, an assembly of neurons transitions to the upstate, enabling the asynchronous, specified action to emerge (Stern et al., 1998). However, in HD, the bidirectional cortico-striatal network is no longer able to precisely orchestrate action as nested frequencies uncouple (Miller et al., 2011). Together with the thalamocortical network, which helps set the membrane potential of the MSNs, the system becomes deregulated. As redundancies in the system also dissociate, the behavior chunk fails.

This high degree of coordination needed to facilitate a desired movement is evident when studied on a smaller scale. In mouse models of HD, widespread electrophysiologic dysfunction at the neuronal level is observed. The MSNs demonstrate a depolarized resting membrane potential. This induces hyper-excitability of the depolarization dependent NMDA receptor in response to glutamate. The overactive cells create an energy sink that may lead to cellular death (Rebec et al., 2006). Similar findings are noted in cortical pyramidal cells. As the disease progresses, the overactive cortico-striatal pathway eventually becomes less active and cortical synaptic inputs are lost (Cepeda et al., 2003).

Normalizing large-scale nested oscillations or stabilizing more localized dysfunctional units in HD is challenging. To achieve this, establishing how fundamental frequencies within the striatum evolve throughout the course of HD will be paramount. Recordings from the dorsal striatum in one study of freely moving normal rats demonstrated LFPs in the 1–30 Hz range with a relatively isolated peak at 50–55 Hz (Masimore et al., 2004). Still, LFPs in HD patients have not been well characterized (Estrada-Sánchez and Rebec, 2013). Once known, an attempt to tune the local frequency with electrical stimulation (ES) to regulate function may be possible.

In HD, re-entrainment of the striatum through ES of both the direct and indirect pathway, may repair learning deficits or reduce the rate of loss of existing habitual circuits. ES may also be able to partially restore the energy imbalance by reducing the hyper-excitable state, consequently preserving cortical inputs. Interestingly, in some animal models of HD, the cortex assumes a hyper-excitable state only after the disease has progressed. This finding contrasts with the observations in MSNs but may mark an identifiable time point for intervention (Cummings et al., 2009).

## Deep Brain Stimulation for HD

There are several reports describing the use of DBS in HD when debilitating chorea predominates in the presence of atrophy and structural changes. We must be cautious in the interpretation of these studies based on the heterogeneity (Table 1). The target most often selected was GPi and DBS was primarily offered to control medically refractory chorea in most cases; although the progression to hypokinesia later in the disease can sometimes complicate interventions (Reiner, 2004). Although cognition was either not addressed or mentioned only in brief, these studies serve as a starting point for the next generation of DBS therapies for HD.

**Table 1:** **Summary of published deep brain stimulation reports for HD**.

	Target(s)	*N*	Ages(s) at surgery (years)	Approximate disease duration prior to DBS (years)	CAG repeat length(s)
**Moro et al. (2004)**	GPi	1	43	8	-
**Hebb et al. (2006)**	GPi	1	41	13	47
**Biolsi et al. (2008)**	GPi	1	60	10	44
**Fasano et al. (2008)**	GPi	1	72	17	-
**Ligot et al. (2011)**	GPe	5	41–60	2–5	41–53
**Kang et al. (2011)**	GPi	2	57	10	42
			50	5	41
**Groiss et al. (2011)**	GPi	1	65	-	-
**Spielberger et al. (2012)**	GPi	1	30	9	58
Cislaghi et al. (2013)*	GPi	1	27	12	74
**Huys et al. (2013)**	GPi	1	40	3	-
**Velez-Lago et al. (2013)**	GPi	2	34	7	60
			25	6	68
**Gonzalez et al. (2014)**	GPi	7	30–78	3–8	40–50
**Gruber et al. (2014)**	STN and GPi	1	41	9	49
**Beste et al. (2014)**	GPe	2	57	-	42
			32		53

*Westphal variant; (-) indicates data was not found or reported.

In one of the earliest published reports, bilateral GPi leads were implanted in a patient with pharmacologically-refractory HD chorea (Moro et al., 2004). 40 Hz stimulation improved the chorea and dystonia in this patient. The effect was enhanced at higher frequencies (130 Hz) but this exacerbated the bradykinesia, corroborating findings in other studies. Positron Emission Tomography (PET) studies in this patient also demonstrated increased cerebral blood flow in the supplementary motor area, anterior cingulate cortex and sensorimotor cortex with “ON” stimulation only. It is unclear what, if any, cognitive gains may have been facilitated by DBS. In the wake of the success treating this initial patient, others followed, publishing their findings after bilateral GPi DBS for HD with mixed, but generally favorable, results (Hebb et al., 2006; Spielberger et al., 2012).

Biolsi et al. described a patient with bilateral GPi DBS implantation for chorea, which also had moderate subcortical cognitive dysfunction at the time of surgery (Biolsi et al., 2008). Four years later, the cognitive dysfunction remained stable and the patient was still able to perform complicated motor tasks. DBS in this case reduced the chorea and may have been neuroprotective, although his disease progression had been noted to be slow even prior to DBS. In another case, bilateral GPi DBS was performed which demonstrated global progressive cognitive decline on serial testing. He, like others, developed bradykinesia after DBS that prevented stable ambulation (Fasano et al., 2008). In two other patients who underwent bilateral GPi, chorea was improved at 2 years but cognitive decline continued, suggesting DBS was unable to halt progression of cognitive dysfunction (Kang et al., 2011).

Another report of two patients who underwent DBS of the GPi as a palliative treatment for disabling motor symptoms was recently published (Velez-Lago et al., 2013). In one patient, for whom chorea was the predominate symptom, DBS reduced a chorea subscore on the Unified HD Rating Scale. In the second patient, DBS was offered as a treatment of generalized, non-fixed dystonia. This failed to improve her dystonia, and the devices were eventually turned off. Although GPi DBS is a well-established treatment for primary generalized dystonia, the dystonic symptoms in HD are unique and appear to reflect the loss of direct pathway striatal neurons in advanced disease.

In the largest published series to date, seven patients with refractory chorea were treated with bilateral GPi DBS between 2008 and 2010 (Gonzalez et al., 2014). Similar to the other studies, chorea improved on average 60% at 1 year but a favorable improvement on the total motor score was not observed (Gonzalez et al., 2014). Importantly, DBS did not alleviate dystonia; while deactivation of stimulation reactivated chorea, indicating that, as for other movement disorders, the effects on motor symptoms depend on continuous stimulation.

Gruber et al. implanted a patient using simultaneous STN and GPi DBS in an attempt to recalibrate the direct and indirect pathways. This strategy minimized the bradykinesia observed following GPi stimulation alone (Gonzalez et al., 2014). Not surprisingly, when STN was used alone, chorea was not sustainably improved (Gruber et al., 2014).

Because of its role in error processing control, the GPe is another potential target for DBS in patients with HD exhibiting cognitive dysfunction. Error processing control, a measure of negative feedback that updates a behavioral action in real time, is believed to underlie early executive dysfunction in HD (Smith et al., 2000). Temel et al. tested DBS of the globus pallidus externus in a transgenic rat model of HD to evaluate the effect on motor and cognitive function (Temel et al., 2006). Cognitive function was measured by performance on a choice reaction time test. Transgenic rats will prematurely release a lever associated with a food reward compared to controls. This indicates that transgenic rats are unable to suppress the unwanted response. In both wild type rats and transgenic rats, DBS improved performance on the choice reaction time test relative to preoperative testing. Although the number of animals tested was small, this study suggests DBS restores unwanted response inhibition in the indirect pathway—a necessary precursor for executing learned behavior in the co-activation model.

In part, based on these results, Ligot et al. proposed that inhibitory stimulation of GPe would suppress thalamocortical hyperactivity-induced hyperkinesia as well as influence cognitive and behavioral symptoms (Ligot et al., 2011). To study this effect, the investigators used PET imaging to compare 15 control subjects with 5 HD patients implanted with stimulation electrodes in GPe. In the resting state, cortico-subcortical regional cerebral blood flow is reduced in HD with the stimulator off. In keeping with the basal ganglia thalamocortical circuit model, GPe stimulation modulated connectivity and further reduced regional cerebral blood flow in the basal ganglia, cortical structures and the default mode network (Ligot et al., 2011). These findings lead to the conclusion that stimulation of GPe may benefit patients with HD. In a recently completed prospective pilot study, intended to test both GPe and GPi stimulation using a crossover design, DBS of GPe in two patients with HD seemed to restore error processing control, although this effect was washed out when the system was turned off (Beste et al., 2014). Based on these studies and current models of learning, it is our impression, that GPe DBS warrants continued study as a potentially therapeutic target in HD.

## Deep Brain Stimulation for HD: Future Directions

The effects of HD lead to cortical disassociation where new behaviors and motor tasks are no longer acquired and existing concatenations that subserve simple and complex skills are decoupled. While the current results of DBS in HD patients may seem disappointing at first, a great deal has been learned. Earlier human studies are primarily aimed at evaluating safety of a novel therapy. The current stage of knowledge seems to support that GPi and GPe DBS may or may not improve motor symptoms but, in general, effects of stimulation were not deleterious. With safety largely established, new studies can be initiated including controlled trials. New therapies could be aimed at realigning cortical and subcortical structures or reinforce existing pathways, particularly when neural compensation is most responsive (Papoutsi et al., 2014). Cognitive biomarkers that can be detected during the prodromal stages and followed during disease progression, such as working memory, psychomotor speed, reaction to negative emotions, and executive functioning, may be most promising (Dumas et al., 2013).

**Figure 1:**
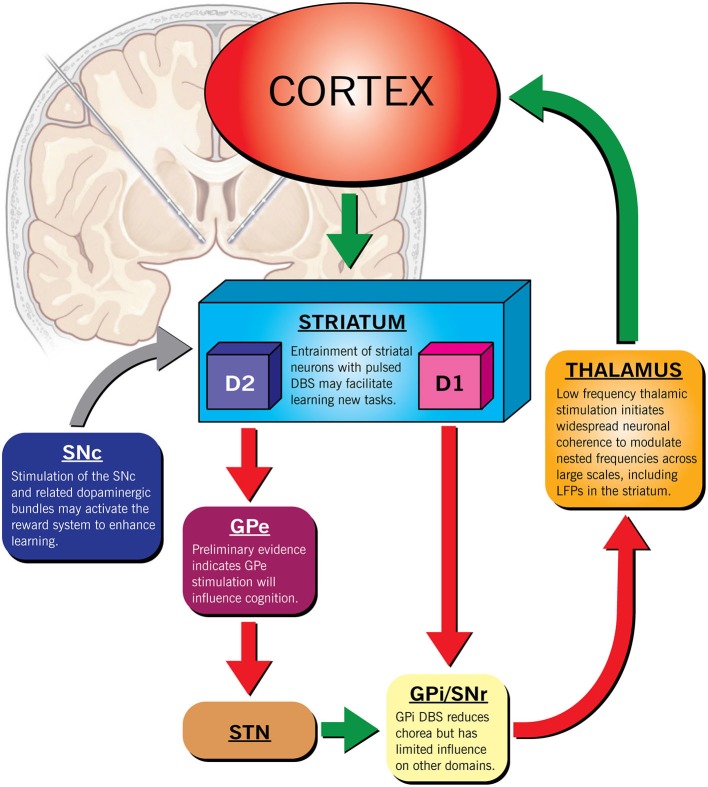
**Therapeutic targets for deep brain stimulation in HD**. The scale of the response could be modulated by using different stimulation paradigms in addition to the location of the lead(s). For example, increasing dopaminergic activity through SNc stimulation may improve non-specific striatal processing that governs learning. Similarly, thalamic stimulation at low frequencies may modulate multiple nested frequencies simultaneously to re-establish a normal frequency spectrum. High frequency, triggered focal, pulsed stimulation of the striatum paired to a specific task could augment learning of specific skills by boosting event sequencing.

It is our opinion that DBS may hold promise in preserving brain function in patients with HD to maintain independence and reduce suffering. However, because the phenotypic expression of HD is heterogeneous, DBS may only benefit a select group of patients. Determining this subset is paramount to the success of any intervention. Fortunately, recent large-scale longitudinal studies (such as PREDICT-HD) that have identified specific functional imaging signals indicating early neuronal dysfunction in HD have exposed this window of opportunity. Targeted ES, early in the course of disease may be able to overcome limitations via several mechanisms including: (1) regulation of dopamine homeostasis, (2) focal inhibition or (3) activation of local brain regions, (4) task specific, triggered closed-loop stimulation or (5) diffuse modulation of multiple pathways with low frequency stimulation (Figure 1). Deciding when to initiate a disease modifying therapy in HD is challenging. Intervention in the prodromal stage exposes an individual to risk when they may be able to live and work independently for many more years. Similarly, initiating therapy after significant cell death, a precursor to end-stage disease, may be futile. The ethical considerations in delivering neuromodulation should therefore be taken very seriously before such an intervention is delivered to a desperate population (Ford and Kubu, 2006).

The question remains open on whether DBS may have any effect in slowing disease progression in HD. To date DBS has not demonstrated neuroprotection for motor decline in other degenerative conditions (Lilleeng et al., 2014). We believe HD may be unique to PD in the ability to identify gene positive individuals prodromally and closely predict the age of motor symptom onset, early in the course of cortico-striatal dysfunction. Furthermore, even if unable to change the trajectory of a terminal disease such as HD, the ability to successfully improve functional capacity and, in turn, optimism (especially early in the disease process), would have a substantial impact on quality of life at an individual level, and the potential for positive economic consequences at a societal level.

## Author Contributions

Conception (SN, AM, MP); Drafting and/or revision (SN, AM, JG, DL, MP); Final approval (SN, AM, JG, DL, MP); Agreement to accountability (SN, AM, JG, DL, MP).

## Conflict of Interest Statement

Andre Machado, MD declares the following:

Distribution rights from Intellectual Property for the following entities: Enspire, ATI and Cardionomics.

Chief Scientific Officer, Enspire.

Consultant: Functional Neuromodulation and Spinal Modulation.
